# Crowdsourcing the General Public for Large Scale Molecular Pathology Studies in Cancer

**DOI:** 10.1016/j.ebiom.2015.05.009

**Published:** 2015-05-09

**Authors:** Francisco J. Candido dos Reis, Stuart Lynn, H. Raza Ali, Diana Eccles, Andrew Hanby, Elena Provenzano, Carlos Caldas, William J. Howat, Leigh-Anne McDuffus, Bin Liu, Frances Daley, Penny Coulson, Rupesh J. Vyas, Leslie M. Harris, Joanna M. Owens, Amy F.M. Carton, Janette P. McQuillan, Andy M. Paterson, Zohra Hirji, Sarah K. Christie, Amber R. Holmes, Marjanka K. Schmidt, Montserrat Garcia-Closas, Douglas F. Easton, Manjeet K. Bolla, Qin Wang, Javier Benitez, Roger L. Milne, Arto Mannermaa, Fergus Couch, Peter Devilee, Robert A.E.M. Tollenaar, Caroline Seynaeve, Angela Cox, Simon S. Cross, Fiona M. Blows, Joyce Sanders, Renate de Groot, Jonine Figueroa, Mark Sherman, Maartje Hooning, Hermann Brenner, Bernd Holleczek, Christa Stegmaier, Chris Lintott, Paul D.P. Pharoah

**Affiliations:** a Department of Oncology, University of Cambridge, Cambridge, UK; b Department of Gynecology and Obstetrics, Ribeirao Preto School of Medicine, University of Sao Paulo, Ribeirao Preto, Brazil; c Department of Physics (Astrophysics), University of Oxford, Oxford, UK; d Cancer Research UK, Cambridge Institute, Cambridge, UK; e University of Southampton, Southampton, UK; f University of Leeds, Leeds, UK; g Addenbrookes Hospital NHS Trust, Cambridge, UK; h Institute of Cancer Research, London, UK; i Cancer Research UK, London, UK; j Netherlands Cancer Institute, Antoni van Leeuwenhoek Hospital, Amsterdam, The Netherlands; k Department of Public Health and Primary Care, University of Cambridge, Cambridge, UK; l Human Genotyping (CEGEN) Unit, Human Cancer Genetics Program, Spanish National Cancer Research Centre (CNIO), Madrid, Spain; m Biomedical Network on Rare Diseases (CIBERER), Madrid, Spain; n Cancer Epidemiology Centre, Cancer Council Victoria, Melbourne, Australia; o Centre for Epidemiology and Biostatistics, Melbourne School of Population and Global Health, The University of Melbourne, Australia; p Institute of Clinical Medicine, Pathology and Forensic Medicine, University of Eastern Finland, Finland; q Department of Laboratory Medicine and Pathology, Mayo Clinic, Rochester, MN, USA; r Department of Human Genetics & Department of Pathology, Leiden University Medical Center, Leiden, The Netherlands; s Department of Surgical Oncology, Leiden University Medical Center, Leiden, The Netherlands; t Sheffield Cancer Research, Department of Oncology, University of Sheffield, Sheffield, UK; u Academic Unit of Pathology, Department of Neuroscience, University of Sheffield, Sheffield, UK; v National Cancer Institute, USA; w Department of Medical Oncology, Family Cancer Clinic, Erasmus MC Cancer Institute, Rotterdam, The Netherlands; x Division of Clinical Epidemiology and Aging Research, German Cancer Research Center (DKFZ), Heidelberg, Germany; y Division of Preventive Oncology, German Cancer Research Center (DKFZ), Heidelberg, Germany; z German Cancer Consortium (DKTK), German Cancer Research Center (DKFZ), Heidelberg, Germany; aa Saarland Cancer Registry, Saarbrücken, Germany

**Keywords:** Citizen science, Crowd science, Crowdsourcing, Breast cancer

## Abstract

**Background:**

Citizen science, scientific research conducted by non-specialists, has the potential to facilitate biomedical research using available large-scale data, however validating the results is challenging. The Cell Slider is a citizen science project that intends to share images from tumors with the general public, enabling them to score tumor markers independently through an internet-based interface.

**Methods:**

From October 2012 to June 2014, 98,293 Citizen Scientists accessed the Cell Slider web page and scored 180,172 sub-images derived from images of 12,326 tissue microarray cores labeled for estrogen receptor (ER). We evaluated the accuracy of Citizen Scientist's ER classification, and the association between ER status and prognosis by comparing their test performance against trained pathologists.

**Findings:**

The area under ROC curve was 0.95 (95% CI 0.94 to 0.96) for cancer cell identification and 0.97 (95% CI 0.96 to 0.97) for ER status. ER positive tumors scored by Citizen Scientists were associated with survival in a similar way to that scored by trained pathologists. Survival probability at 15 years were 0.78 (95% CI 0.76 to 0.80) for ER-positive and 0.72 (95% CI 0.68 to 0.77) for ER-negative tumors based on Citizen Scientists classification. Based on pathologist classification, survival probability was 0.79 (95% CI 0.77 to 0.81) for ER-positive and 0.71 (95% CI 0.67 to 0.74) for ER-negative tumors. The hazard ratio for death was 0.26 (95% CI 0.18 to 0.37) at diagnosis and became greater than one after 6.5 years of follow-up for ER scored by Citizen Scientists, and 0.24 (95% CI 0.18 to 0.33) at diagnosis increasing thereafter to one after 6.7 (95% CI 4.1 to 10.9) years of follow-up for ER scored by pathologists.

**Interpretation:**

Crowdsourcing of the general public to classify cancer pathology data for research is viable, engages the public and provides accurate ER data. Crowdsourced classification of research data may offer a valid solution to problems of throughput requiring human input.

## Highlights

• Crowdsourcing of the general public to classify estrogen receptor in breast tumors was evaluated.
• More than 95 thousand volunteers participated of the project over two years.
• Citizen Scientists were able to classify estrogen receptors with high accuracy.
• The estrogen receptor score assigned by Citizens Scientists was associated with breast cancer patient survival.

## 1 Introduction

The assessment of tissue protein expression by immunohistochemistry (IHC) is widely used in both the clinical and the research settings. IHC combined with tissue microarray (TMA) technology (Wan et al., 1987, Kononen et al., 1998) provides an efficient approach to the study of multiple molecular markers in hundreds or thousands of tumors. TMAs are produced by removing cylindrical cores of tissue from up to donor paraffin blocks and embedding these into a single recipient paraffin block at set array coordinates. Several hundreds of tumors may be embedded in a single TMA. This has the potential to reduce inter-assay variability and to reduce the cost of research (Camp et al., 2008). Consequently, the large sample sizes required for robust inference in clinical epidemiology are achievable. A typical study may include over 10,000 cases (Ali et al., 2014). However, the process still relies on manual scoring of labeled sections by trained researchers. This is time consuming and scoring remains a rate-limiting step in this type of research. One solution to this bottleneck is to scan the labeled sections and to use automated analysis of the digitized images of each core. Several image analysis algorithms have been shown to perform reasonably well for some IHC markers (Giltnane and Rimm, 2004, Bolton et al., 2010, Ali et al., 2013, Howat et al., 2015). While automated image analysis remains promising, its implementation may be complex and it has not yet replaced manual scoring in large scale molecular epidemiology studies in cancer.

An alternative approach to automated image analysis is crowdsourcing in which a function – here scoring of IHC labeled sections of tumor cores – is outsourced to an undefined and generally large group of people in the form of an open call. The crucial prerequisites are the use of the open call format and the large network of potential contributors (Howe, 2006). Crowdsourcing relies on parallel independent inputs from individuals allowing for large group size, maximizing cognitive diversity and enhancing group performance (Page, 2008).

The Citizen Science Alliance (http://www.citizensciencealliance.org) is a collaboration of scientists, software developers and educators, who use the concept of crowdsourcing to develop, manage and utilize internet-based citizen science projects in order to further scientific research and to promote the public understanding of science. Through citizen science projects, thousands of Citizen Scientists have collected, organized and classified data for research purposes. Some successful initiatives are: the investigation of galaxy morphology (Lintott et al., 2008), the prediction of protein structures (Cooper et al., 2010) and the alignment of multiple sequences in genomic studies (Kawrykow et al., 2012). The Cell Slider project was established to enable the scoring of tumors labeled using IHC by untrained members of the general public – Citizen Scientists – through an internet-based interface. In this paper we report the results of the first Cell Slider project in which Citizen Scientists scored estrogen receptor (ER) expression in images of tumor cores from a large number of breast cancers arrayed in TMAs.

## 2 Methods

### 2.1 Study Design, Setting, and Population

**Fig. 1:**
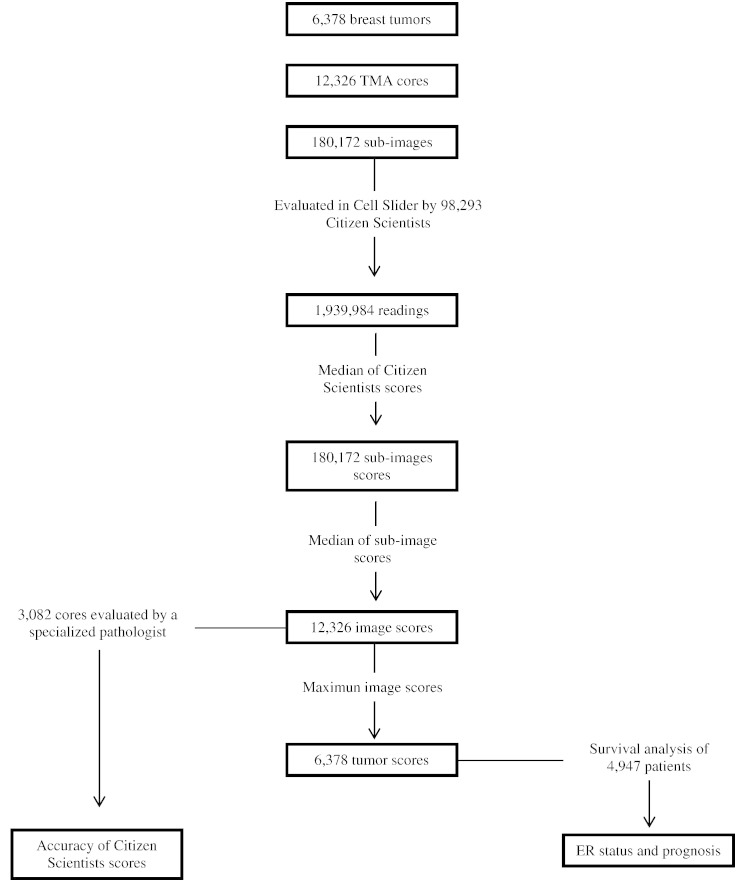
Flowchart of study design showing details of score calculation (sub-image, image and tumor), comparison with pathologist evaluation and survival analysis.

This study was performed using pathology data from the Breast Cancer Association Consortium (BCAC), an international collaboration that was established to provide large sample sizes for examining risk factors, genetic associations and prognostic markers in breast cancer (Breast Cancer Association Consortium, 2006). The BCAC resource comprised 12,326 scanned images from breast cancer TMA cores stained for estrogen receptor (ER). A total of 3082 cores from the SEARCH study (Lesueur et al., 2005) — that had been previously been scored by the same pathologist under conventional microscopy and without access to patient clinical records. The cores were from the tumors of 6378 patients from 10 studies (Appendix 1). Information on clinic-pathological characteristics of each patient was obtained from clinical records or centralized review of case notes. This included ER status for which was either taken from independent research-based pathology review or, where this had not been carried out, from the clinical records. Relevant research ethics committees approved all the studies and samples were anonymized before being sent to two coordinating centers at Strangeways Research Laboratory (University of Cambridge, UK) and the Breakthrough Pathology Core Facility (Institute of Cancer Research, London, UK) for analysis. Fig. 1 summarizes the study design.

TMA sections were immunostained in several centers and each stained TMA slide was digitized using the Ariol platform (Genetix Ltd, Hampshire, UK) and high-resolution images or each tumor core were subsequently extracted for analysis. The ease of scoring of TMA images before and after transforming the colors in a variety of combinations was evaluated by beta-testing by experienced Citizen Science Alliance volunteers. The preferred colors were then used subsequently. The colors of the images were transformed using the ImageMagick library. The colors of the image were first negated (replacing each pixel by its complementary color) and then the saturate was increased by 300% and the hue reduced by 82%. Finally the full image was divided into 16 sub-images which were resized to 495 by 496 pixels each. The four corner sub-images were removed as they often had no tumor material present and the remaining 12 sub-images were uploaded to the Cell Slider project web site.

### 2.2 Citizen Scientist Training and Scoring

Any member of the public (Citizen Scientists) can participate of the project at http://www.cellslider.net/. Once in the website the Citizen Scientist can register a user name and a password or proceed without registration. At first entry the Citizen Scientist is provided with a brief web based training tutorial in which the task and key steps required to score each image are described. After completing the training the Citizen Scientist is presented with an image to score, which is done by clicking on the answers for up to four questions presented serially (Supplemental figure 1). The Citizen Scientist is first asked to identify the presence of cancer cells in the sub-image (yes/no). If they do identify cancer cells, the Citizen Scientist is asked to estimate: the number of cancer cells on a scale of 1 to 4 corresponding to 1 to 5, 6 to 10, 11 to 20 and more than 20 cancer cells; the proportion of nuclei staining positive on a scale of 0 to 5 corresponding to 0%, < 1%, 1 to 9%, 10 to 33%, 34 to 66% and 67 to 100%; and the intensity of staining on a scale of 0 to 3 corresponding to no staining, weak, moderate and strong staining. This scoring was designed to approximate the Allred scoring system which is commonly used in clinical practice (Howat et al., 2015). The Allred system scores the proportion of nuclei staining positive on a scale of 0–5 (0%, < 1%, 1 to 9%, 10 to 33%, 34 to 66% and 67 to 100%) and the intensity of staining on a four point scale (no staining, weak, moderate and strong, 0–3). These scores are added together to provide an overall score on a nine point scale (0 to 8). A tumor with an Allred score > 2 is conventionally considered to be ER positive. A pseudo-Allred score for each Citizen Scientist sub-image evaluation was generated by summing the proportion score with the intensity score.

### 2.3 User Performance Score

A set of 200 sub-images, selected to provide a range of scores, were accessed and scored by a pathologist. This set of sub-images was used as a standard to generate a user performance score (UPS) for each Citizen Scientist according to their performance in identifying cancer cells in the sub-images as follows: (1) All of the Citizen Scientists are assigned a preliminary UPS of 0.5 and the experts are assigned a UPS of 1 on initializing the algorithm. (2) The preliminary UPSs of the Citizen Scientists who have scored a sub-image that was scored by an expert are then increased to 0.7 if the Citizen Scientist agreed with the expert otherwise the preliminary UPS remains as 0.5. (3) A modal classification (a pseudo-likelihood that the image contains cancer) is then generated for all the sub-images without an expert classification as the weighted average of all the sub-image scores where a classification of cancer cells present is scored as one and no cancer cells present is scored as zero. The weights are the Citizen Scientist preliminary UPSs as assigned in (2). (4) The UPS is then recalculated for each user based on the modal classification for all the sub-images they have scored. If the user identifies cancer cells and the modal classification value is > 0.5 their score for that sub-image is the modal value. If the user does not identify cancer cells and the consensus value is < 0.5 their score for that sub-image is 1 — the modal value. If the user disagrees with the modal classification (cancer cells present and the modal value < 0.5 or cancer cells not present and the modal value > 0.5) the user is assigned a score of 0 for that sub-image. The recalculated UPS is then the average user score for all images they have scored. (5) The average change in mean UPS from (2) to (3) is then calculated. If this value is greater than 0.0003 then (3) and (4) are repeated until the change in average UPS is less than 0.0003 and the final UPS is assigned to each user. This approach is an efficient method for obtaining a UPS without the need for each Citizen Scientist to score sub-images that have already been scored by a pathologist.

### 2.4 Image Score

A single score for each image (tumor core) was obtained by first combining the scores for each sub-image to generate a single sub-image score and then combining the 12 sub-image scores. Three different approaches were used to combine the data for multiple scorers to generate a single score for each sub-image: i) the median of individual readings, ii) the weighted median of individual scores using the Citizen Scientist user performance score as the weight, iii) the median of individual readings after excluding scores of Citizen Scientists who had scored fewer than five sub-images. The sub-image scores were then combined to obtain a single score for each image by calculating a weighted median of the sub-image scores using the number of cancer cells as the weight. To generate the number of cancer cells score for each image, we calculated the sum of the sub-image median for the number of cancer cells based on the five-point ordinal scale described above. A pseudo-Allred score (range: 0 to 8) for each image was generated by combining the scores for each sub-image to generate a sub-image score and then combining the sub-image scores. (1) The pseudo-Allred score for each sub-image was the median of the individual pseudo-Allred scores; (2) the pseudo-Allred score for the image was then the weighted median of the sub-image scores using the number of cancer cells as the weight.

### 2.5 Statistical Analysis

Spearman's correlation coefficient was used to assess correlations between the Citizen Scientist's performance score and the number of cores scored by each Citizen Scientist and between the Citizen Scientist's ER scores for each TMA core and the Allred score assigned by a pathologist. The receiver-operator characteristic (ROC) was used to evaluate the accuracy of Citizen Scientists' classification of the presence of cancers cells in a tumor core and classification of ER status. Survival time analysis was conducted using Kaplan Meier survival curves and Cox proportional hazards regression. The multivariable Cox proportional hazards model was stratified by study and included ER status, patient age, tumor stage and tumor grade. Estrogen receptor status is known to violate the Cox proportional hazards assumption (Blows et al., 2010) and so ER status was treated as a time-varying covariate in the Cox models in which the ER status specific hazard ratio was assumed to vary linearly with the natural logarithm of time. Stage and grade were missing for 7.8% and 9.3% of cases respectively. We used multiple imputations by chained equations to deal with missing data as this has been shown to be the method of dealing with missing data that is least likely to bias parameter estimates (Ali et al., 2011, Graham et al., 2007, White et al., 2011). Each data set was imputed 20 times and the parameter estimates from the Cox regression models were combined using Rubin's rules (Rubin, 1976). Statistical analysis was conducted using STATA/SE version 13 (StataCorp).

## 3 Results

### 3.1 Citizen Scientists Participation

**Fig. 2:**
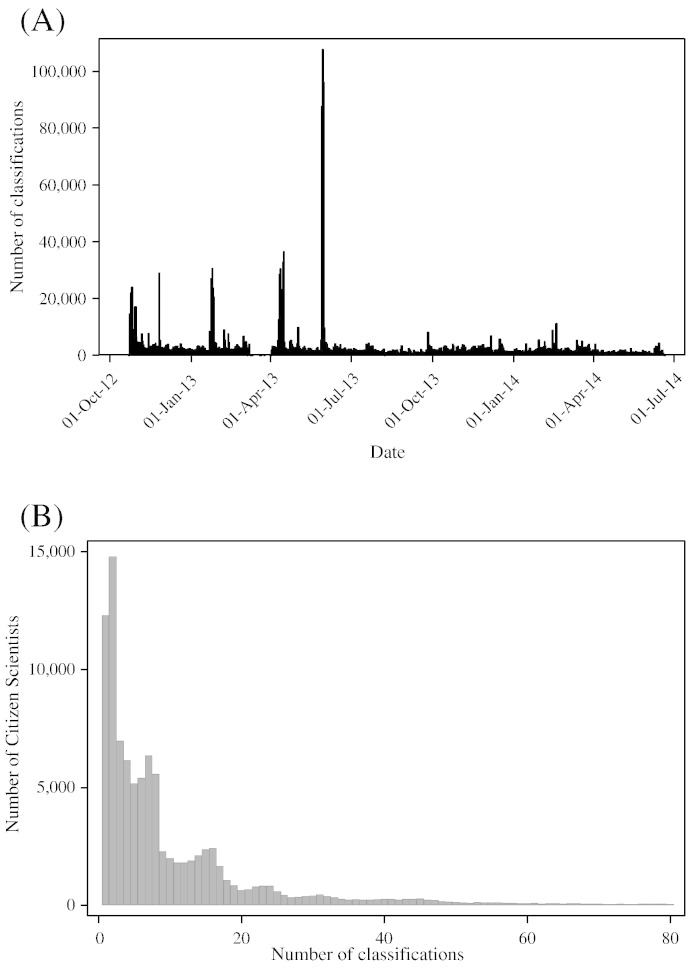
Engagement in Cell Slider by Citizen Scientists. (A) Number of classifications by day since project launch; (B) histogram of number of classifications done by each Citizen Scientist.

Cell Slider was launched on 23 October 2012. The press release was picked up by several media outlets, with coverage in the Huffington Post and on the UK terrestrial television channel ITV. From October 2012 to June 2014, 98,293 Citizen Scientists accessed the Cell Slider web page and scored 180,172 sub-images derived from images of 12,326 stained TMA cores (Fig. 2A). A further article on Reddit.com on 25 November 2012 resulted in over 15,000 additional visitors within a short time. Subsequent media exposure and advertising on Facebook resulted in three spikes of classifications in 2013 with a peak of 107,710 on 30th May 2013, but the effect was generally short-lived; after a few days, user numbers returned to a few hundred per day. A total of 1,939,984 sub-image classifications were available for analysis. The median number of sub-images evaluated by each citizen scientist was 6 (5th percentile = 1; 95th percentile = 47; Fig. 2B). The distribution of the number of scores for each sub-image is bimodal: there were five scores for 46% of sub-images and 20 scores for 29% of the sub-images. The total number of sub-image scores for each image was also bimodal and ranged from 60 to 361, with 64% having 60 to 157 scores (median = 70) and 36% having 251 to 361 (median = 306) Citizen Scientists.

### 3.2 Citizen Scientists Performance

**Fig. 3:**
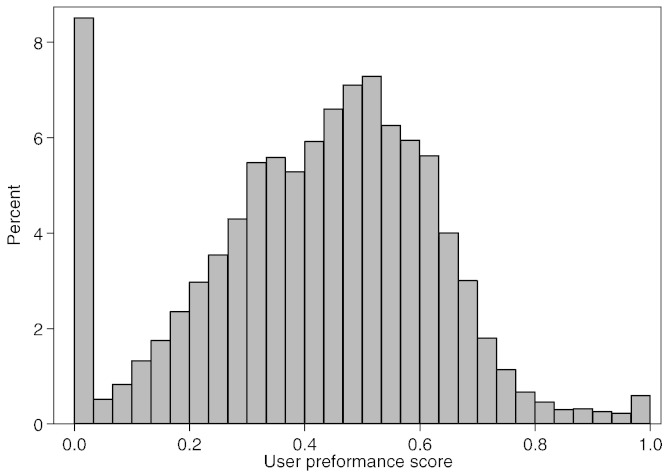
Histogram of user performance final scores.

We first assessed the individual performance of Citizen Scientists in identifying the presence of cancer cells in sub-images based on the final UPS. The distribution of the Citizen Scientist UPSs is shown in Fig. 3. The UPSs were weakly correlated with the number of images scored (Spearman rho = 0.26, P < 0.0001). Eight percent of the Citizen Scientists (7835) were assigned a UPS of zero. Most of these (80%) had scored only one or two sub-images.

**Fig. 4:**
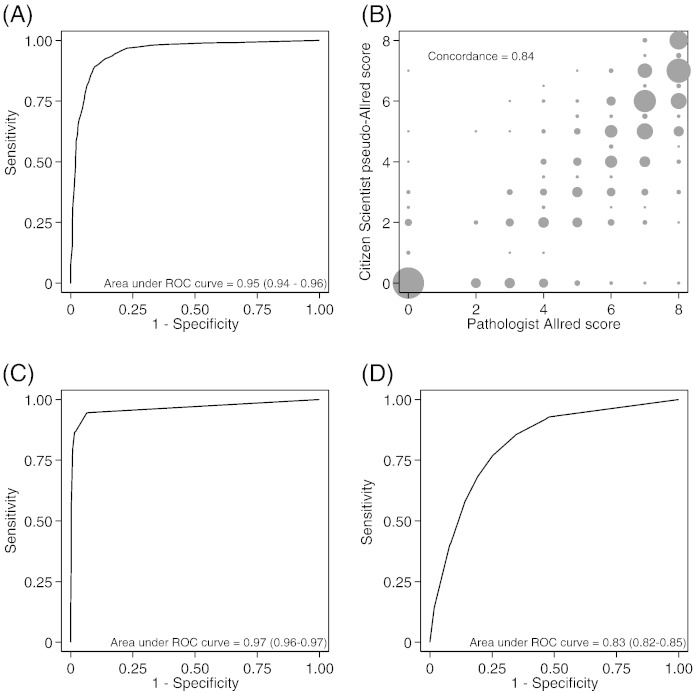
Accuracy of Citizen Scientist classifications. (A) Receiver operator characteristic curve for identification of cancer cells by Citizen Scientists in 3038 tumors from the SEARCH study compared to classification by a pathologist. (B) Frequency weighted scatterplot of Citizen Scientist pseudo-Allred score against the pathologist assigned Allred score for 2121 tumors from the SEARCH study. (C) Receiver operator characteristic curve for classification of ER status based on Citizen Scientist pseudo-Allred score against the pathologist classification for 2121 tumors from the SEARCH study; (D) receiver operator characteristic curve for classification of ER status based on Citizen Scientist pseudo-Allred score against the ER status recorded in the Breast Cancer Association Consortium data base from a variety of sources for 10,679 tumors from ten studies.

We evaluated the accuracy of the Citizen Scientists in identifying the presence or absence of cancer cells using data from a subset of the TMA cores – 3082 from the SEARCH study (Lesueur et al., 2005) – that had been previously scored by a pathologist. This took 10 h of pathologist time. The pathologist identified cancer cells in 2138 (69%) of these cores. The number of cancer cells was calculated for each image as the sum of the median number of cancer cells scored by the Citizen Scientists for each sub-image. This score ranged from 0 to 64 across the 3082 images. A threshold of at least one cancer cell correctly classified 2121 of the 2138 cores containing cancer cells according to the pathologist (sensitivity 99%) and 296 of the 944 cores with no cancer cells (specificity 31%); overall 78% of all classifications were correct. The receiver operator characteristic curve plots the sensitivity against 1 minus the specificity at different cut-offs for classification of cancer cells present or absent (Fig. 4A). The area under the ROC curve was 0.95 (95% CI 0.94 to 0.96). Maximum accuracy was achieved using a threshold of at least ten cancer cells to classify tumor cores as cancer cells present, which resulted in a sensitivity of 97%, a specificity of 77% and an overall accuracy 91%.

We then compared the ER staining as measured by the Allred score assigned by the pathologist with the Citizen Scientist pseudo-Allred score for the subset of 2121 cores with cancer cells identified by both the pathologist and the Citizen Scientists. The correlation coefficient was 0.90 (P < 0.0001) and the mean of difference (Pathologist score–Citizen Scientists score) was 1.09 (95% CI 1.04 to 1.15) (Fig. 4B). Under the standard Allred scoring system, a score of > 2 is conventionally classified as ER positive (Harvey et al., 1999). Sixteen hundred and eleven of these tumors were classified as ER positive by the pathologist. Using a cut-off for the pseudo-Allred score of > 2, the sensitivity of ER status determined by the Citizen Scientists was 88%, with a specificity of 98%. There was agreement between pathologist and Citizen Scientists for 1912 tumors (90%). There were nine discordant tumors with pseudo-Allred score > 2 and Allred score ≤ 2 and 200 tumors with pseudo-Allred score ≤ 2 and Allred score > 2. The sensitivity and specificity for classifying ER status using different cut points of the pseudo-Allred score is shown using a ROC curve (Fig. 4C). The area under the ROC curve was 0.96 (95% CI 0.95 to 0.97). ER status (positive or negative) without an Allred score was available for 2842 additional tumors from other BCAC studies. The area under ROC curve for the Citizen Scientists ER classification was 0.83 (95% CI 0.82 to 0.85) (Fig. 4D). The distribution of the pseudo-Allred score by known ER status is shown in table 1.

The pseudo-Allred scores were calculated using three alternative approaches. The results of combined scores from the unweighted median of individual readings were slightly better than those from weighted median of individual scores or those including only Citizen Scientists with five or more scored sub-images (Table 2).

### 3.3 Estrogen Receptor Expression and Survival

**Fig. 5:**
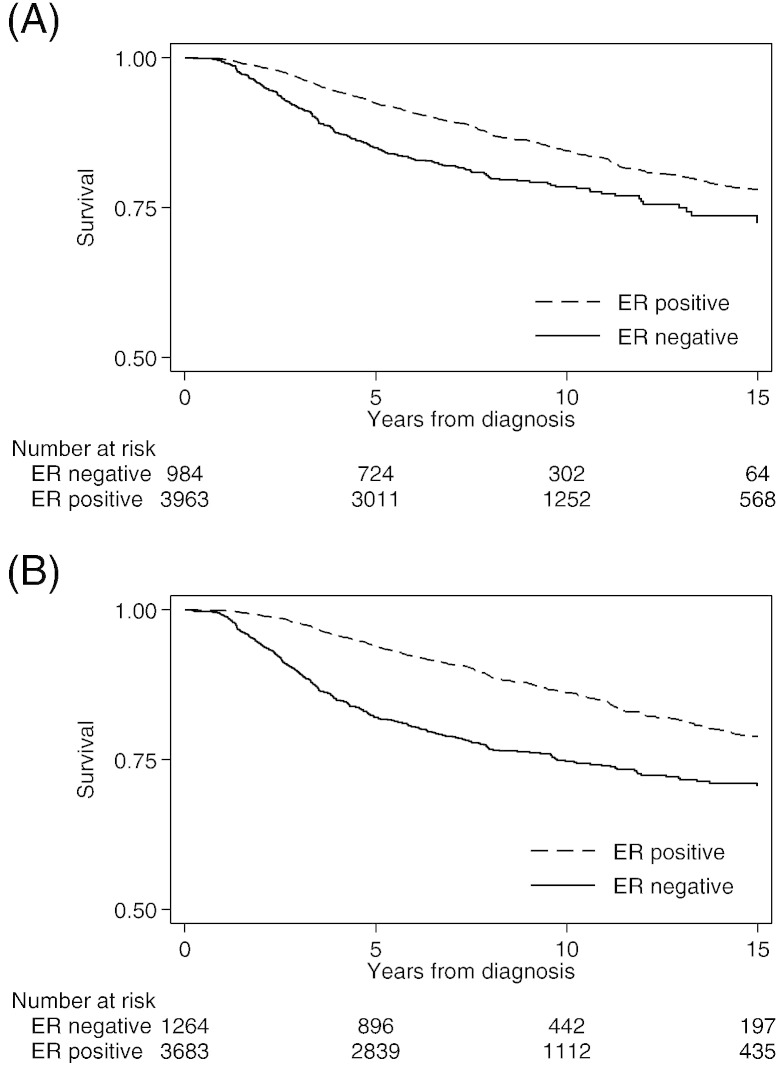
Kaplan–Meier estimates of cumulative survival of 4947 patients. (A) ER status classified by Citizen Scientists. (B) ER status as recorded in BCAC database.

Survival time data were available for 4947 patients in whom there were 734 deaths from breast cancer by fifteen years of follow-up. We used these data to compare the association with prognosis for ER status classified by a pathologist and ER status classified by Citizen Scientists. The Kaplan–Meier survival functions by ER status are shown in Fig. 5. There was a significant association (log rank test P < 0.001) for both determinations of ER status. Based on Citizen Scientists classification, the Kaplan–Meier survival probability estimates at 15 years were 0.78 (95% CI 0.76 to 0.80) for ER-positive and 0.72 (95% CI 0.68 to 0.77) for ER-negative tumors. Based on pathologist classification, survival probability estimates at 15 years were 0.79 (95% CI 0.77 to 0.81) for ER-positive and 0.71(95% CI 0.67 to 0.74) for ER-negative tumors.

We estimated the hazard ratios for breast cancer specific death using Cox regression implemented in a multiple imputation framework to deal with missing data on stage and grade (Table 3). ER status was treated as a time-varying covariate with the log hazard ratio varying linearly with time. This generates two parameter estimates for ER status, β1 and β2, such that the hazard ratio at time t, HR(t), is given by

$$HR\left(t\right)=\exp\left(\beta 1\;+\;\beta 2t\right)\text{.}$$

Based on a comparison of log likelihood statistics, the model using ER status determined by pathologists fit substantially better than that based on the Citizen Scientist data (log likelihoods − 3580.84 and − 3589.38 respectively). The parameter estimates, β1 and β2 were − 1.41 (95% CI − 1.71 to − 1.10) and 0.21 (95% CI 0.16 to 0.26) for the model based on pathologist-determined ER status and − 1.34 (95% CI − 1.69 to − 0.98) and 0.21 (95% CI 0.14 to 0.27) for the model based on Citizen Scientist based ER status. Thus, the hazard ratio for ER positive tumors based on pathologist determination was 0.24 (95% CI 0.18 to 0.33) at diagnosis increasing thereafter to one after 6.7 (95% CI 4.1 to 10.9) years of follow-up. Similarly, the hazard ratio for ER positive scored by Citizen Scientists was 0.26 (95% CI 0.18 to 0.37) at diagnosis and increased thereafter to one after 6.5 (95% CI 4.1 to 12.0) years of follow-up.

## 4 Discussion

The principles of crowdsourcing, which enable sufficiently accurate analysis of a variety of types of scientific data from classifying images of galaxies to categorizing the sounds made by killer whales, are well established. However, it is not self-evident that this approach will be useful for other types of scientific data. We have shown that Citizen Scientists with minimal training can accurately score ER expression in breast tumors.

Assessing individual Citizen Scientist performance is challenging in group aggregate work. In this study, a user performance score was developed to assign weights to Citizen Scientist results according to the level of their agreement with a specialist. However the overall performance of the group was not improved by weighting individual results. This is because of the effective loss of data for those Citizen scientists assigned a weight of zero. Our results agree with previous observations that the average of decisions from a group aggregate is accurate and multiple readings by a large number of individuals can correct for divergent results (Mattingly and Ponsonby, 2014).

In a direct comparison in which the immunohistochemistry staining of over 3000 breast tumors cores arrayed on a TMA were scored by a pathologist and the Citizen Scientists, the Citizens performed well. The major weakness of the Citizen Scientists was in the identification of cancer cells on any given image. In the construction of tissue micro arrays small cores (typically 0.6 mm) of representative areas of tumors are selected. However, the density of cancer cells may vary substantially across a tumor. Consequently, some cores contain no cancer cells at all (Ali et al., 2011). Other cores may include cancer cells but these may be unevenly distributed so that some of the sub-images of a core may not have cancer cells. Citizen Scientists tended to overestimate the presence of cancer cells, probably because of the presence of stained normal cells or technical artifacts. Lymphocyte expression of ER can cause false positive results (Pierdominici et al., 2010). The poor calling of cancer cells is also reported in studies with automated algorithms (Ali et al., 2013, Howat et al., 2015). This limitation is, perhaps, not surprising given that breast cancer cells are morphologically heterogeneous. Furthermore, the pattern recognition skills needed to identify cells with a large nucleus, irregular size and shape, prominent nucleoli, and scarce cytoplasm are greater than those required to simply identify the presence or absence of IHC staining.

The accuracy of the Citizen Scientist classification of estrogen receptor status was extremely good for the subset of images on which there was agreement between pathologist and Citizen Scientist on the presence of cancer cells. The concordance between the pseudo-Allred score assigned by Citizen Scientists and Allred score by the pathologist was 0.84, slightly inferior to the reported concordance between pathologists using the Allred score which varies from 0.87 (Harvey et al., 1999) to 0.90 (Badve et al., 2008). The area under ROC curve was 0.97 (0.96–0.97) for ER status dichotomous classification that was slightly better than the reported value for automated algorithms of 0.92 (0.90–0.94) (Ali et al., 2013). The accuracy of the Citizen Scientists was reduced in the subset of cases for which the comparison was the results of ER status as recorded in the BCAC database. There are several reasons for this difference. The ER status as recorded in the BCAC database was primarily derived from clinical records or central review of cases by individual studies and thus derived from whole tumor sections, whereas the Citizen Scientist scores were based only on the TMA cores. In the presence of within tumor heterogeneity, the whole section and tumor core scores are likely to differ and this is likely to be a particularly problem for those cores containing no cancer cells. Preliminary assessment of each core by a pathologist using rapid visual inspection to identify unsatisfactory cores might improve the performance. However, this would be time consuming, and the added effort to improve scoring would diminish the relative value of crowdsourcing. These effects are likely to be exacerbated in the BCAC data set by the additional variability in tissue preparation from multiple specimen sources.

The group of voluntary Citizen Scientists was constituted through advertising the Cell Slider on several mass media. However this strategy was very successful for recruiting volunteers, the effect was generally short-lived. Two characteristics of Citizen Scientist participation were evident: there were peaks of participation immediately after media advertisement, and the number of participants who scored just few sub-images is high. One important challenge for future projects is how to maintain the participants after the first interaction with the platform.

Another challenge in citizen science projects is the training of participants. The performance of Citizen Scientists was positively associated with the number of readings performed; however the number of readings by each Citizen Scientist was relatively small, with 64% of participants scoring fewer than eight sub-images, and an additional of 19% scored between 9 and 17 sub-images. The training for Cell Slider was very brief and given without any feedback or response to queries. Nor was there any feedback provided to Citizens after they had scored any images. There may be potential to improve the accuracy, particularly for the identification of cancer cells, by using a more comprehensive training. For example, recent evidence has emerged that the training of Citizen Scientists can be improved using video tutorials (Starr et al., 2014). The Cell Slider platform does not allow Citizen Scientists to go back to an image previously scored and update their classification. Because of this limitation, we were not able to evaluate the impact of belief update on the accuracy of Citizen Scientist classification.

Any method used to carry out scoring of immunostaining for large-scale molecular pathology studies will be subject to a degree of measurement error, and all such methods will be sub-optimal compared to review by a single highly-trained pathologist. However the key question is whether other methods are sufficiently accurate to detect important associations (Camp et al., 2008). We have shown that Citizen Scientist scoring of breast cancer cores is sufficiently accurate to detect an association between ER status and prognosis, with effect sizes only slightly attenuated compared to the effects estimated from clinical data. In our study, each Citizen Scientist was random and uncorrelated with others. Therefore, because of the large size of the group, discrepant inputs should cancel each other out.

We have shown that involving the participation of the general public is a very promising approach to reducing a key bottleneck in the conduct of very large molecular pathology studies. Whether or not it has any advantages over automated image analysis needs to be established and further work is required to establish its utility across a range of markers including proteins in other subcellular compartments. This proof-of-principle study demonstrates that crowdsourced research which engages the general public is a viable method of overcoming key bottlenecks in cancer research studies with great potential for wider application.

## 5 Research in Context

There were evidences that citizen scientists can accurately collect data from the environment and classify data collected by high throughput equipment. We have shown that crowdsourced research which engages the general public is a viable method of overcoming key bottlenecks in cancer research studies. Citizen scientists were able to classify estrogen receptor expression in breast tumors with high accuracy. Our data along with current evidence suggest that citizen science has great potential for wider application in cancer research.

## Contributors

Study conception: PDPP, DE, AH, CL.

Cell Slider website development: HRA, AC, AH, SL, EP, RV.

Cell Slider product management: AC, SC, AH, ZH, JO.

Sample collection and primary data generation: FB, HB, JB, AC, CC, FC, PD, DFE, FD, MG-C, MH, AM, RM, MKS, AC, SSC, RAEMT, CS.

Pathology data management: FB, MB, PC, BL, MG-C, WH, L-AMcD, MS, QW.

Database management: SL, JM, AP, MKS, MG-C.

Data analysis: FJCR, SL, JM, PDPP.

Critical review of draft of the manuscript: all authors.

## Conflicts of interest

We declare that we have no conflicts of interest.

## Figures and Tables

**Table 1:** Distribution of pseudo Allred scores according to ER status.

ER status	Pseudo Allred score			
< 2	2–3	4–5	> 5
SEARCH TMAs (2121 tumors)				
ER negative	481 (22.68%)	26 (1.23%)	2 (0.09%)	1 (0.05%)
ER positive	101 (4.76%)	228 (10.75%)	386 (18.20%)	896 (42.24%)
BCAC TMAs (2842 tumors)				
ER negative	265 (9.32%)	177 (6.23%)	127 (4.47%)	200 (7.04%)
ER positive	69 (2.43%)	132 (4.64%)	284 (9.99%	1588 (58.88%)

**Table 2:** Performance of Citizen Scientists to identify cancer cells and classify ER staining.

Identification of cancer cells in SEARCH study	Obs.	ROC area	95% CI
All original scores	3082	0.951	0.943 to 0.960
Scores from CS with 5 or more scores	3082	0.951	0.942 to 0.960
All original scores with UPS-weighting	3082	0.951	0.942 to 0.959

Classification of ER in SEARCH study	Obs.	ROC area	95% CI

All original scores	2121	0.968	0.961 to 0.974
Scores from CS with 5 or more scores	2121	0.967	0.960 to 0.974
All original scores with UPS-weighting	2121	0.965	0.958 to 0.972

Correlation with the pathologist in SEARCH study	Obs.	Spearman rho	95% CI

All original scores	2121	0.898	0.890 to 0.906
Scores from CS with 5 or more scores	2121	0.896	0.888 to 0.904
All original scores with UPS-weighting	2121	0.894	0.885 to 0.902

Classification of ER in BCAC	Obs.	ROC area	95% CI

All original scores	2842	0.822	0.804 to 0.840
Scores from CS with 5 or more scores	2842	0.821	0.803 to 0.839
All original scores with UPS-weighting	2842	0.820	0.802 to 0.838

CS: Citizen Scientists.

UPS: final user performance score.

**Table 3:** Estimated hazard ratios (HR) for all-cause mortality in 4947 breast cancer patients from multi-variable Cox proportional hazards model after multiple imputations of missing data for stage and grade.

Variable	Cox model with ER evaluated by Citizen Scientists			Cox model with ER reported in BCAC data base		
	HR	95% CI	p-Value	HR	95% CI	p-Value
Age	1.00	0.99–1.00	0.998	1.00	0.99–1.00	0.875
Stage						
I			Ref			Ref
II	2.33	1.91–2.84	< 0.001	2.32	1.90–2.83	< 0.001
III	4.97	3.76–6.57	< 0.001	4.97	3.76–6.56	< 0.001
IV	18.84	12.36–28.73	< 0.001	18.39	12.12–27.89	< 0.001
Grade						
1			Ref			Ref
2	1.58	1.24–2.01	< 0.001	1.55	1.22–1.98	< 0.001
3	2.45	1.91–3.13	< 0.001	2.30	1.79–2.97	< 0.001
ER positive	0.26	0.18–0.37	< 0.001	0.24	0.18–0.33	< 0.001
ER positive TVC⁎	1.23	1.15–1.31	< 0.001	1.23	1.17–1.30	< 0.001

⁎ Time varying covariate.

## Supplementary Material

**Supplementary figure 1.** Serial presentation of tumor core sub-images for Citizen Scientist scoring for classification of estrogens receptors in sub-images. **Supplementary Figure 2.** Bimodal distribution of number scores by sub image and by complete image. **Supplementary table 1.** Distribution of tumors, TMA cores, sub-images and evaluations by study. **Supplementary table 2.** Estimated hazard ratios for all-cause mortality in 4,150 complete case breast cancer patients from multi-variable Cox proportional hazards.
