# Supplementary material for: Crowdsourcing the General Public for Large Scale Molecular Pathology Studies in Cancer
Source: eBioMedicine. 2015 May 9;2(7):681–9. doi: 10.1016/j.ebiom.2015.05.009 (PMC4534635; doi:10.1016/j.ebiom.2015.05.009)
Supplement: Supplementary file 1 — Supplementary figure 1. Serial presentation of tumor core sub-images for Citizen Scientist scoring for classification of estrogens receptors in sub-images. Supplementary Figure 2. Bimodal distribution of number scores by sub image and by complete image. Supplementary table 1. Distribution of tumors, TMA cores, sub-images and evaluations by study. Supplementary table 2. Estimated hazard ratios for all-cause mortality in 4,150 complete case breast cancer patients from multi-variable Cox proportional hazards. [file mmc1.docx]

**Supplementary Material**


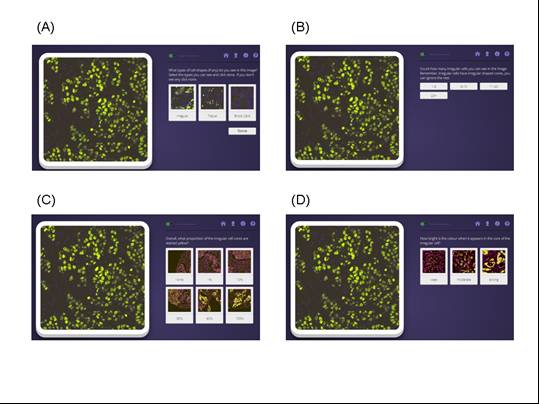


**Supplementary figure 1.** Serial presentation of tumor core sub-images for Citizen Scientist scoring for classification of estrogens receptors in sub-images. **(A)** Identification of tumor cell questions: What types of cell shapes (if any) do you see in this image? Select the types you can see and click done. If you don't see any click none. (Options: Irregular; Tissue; Blood Cells; None). **(B)** Quantification of tumor cells: Count how many irregular cells you can see in the image. Remember, irregular cells have irregular shaped cores, you can ignore the rest. (Options: 1-5; 6-10; 11-20; 20+). **(C)** Quantification of the proportion of positive tumor cells: Overall, what proportion of the irregular cell cores are stained yellow? (Options: 0%; 1%; 10%; 30%; 60%: 100%). **(D)** Classification of the intensity of positive staining: How bright is the colour when it appears in the core of the irregular cell? (Options: weak; moderate; strong).

**Supplementary Figure 2.** Bimodal distribution of number scores by sub image (A) and by complete image (B).

**Supplementary table 1.** Distribution of tumors, TMA cores, sub-images and evaluations by study.

| **Study** | **Tumors** | **%** | **TMA cores** | **%** | **Sub-images** | **%** | **Evaluations** | **%** |
| --- | --- | --- | --- | --- | --- | --- | --- | --- |
| ABCS | 821 | 12.9 | 2,780 | 22.6 | 41,248 | 22.9 | 292,466 | 15.1 |
| CNIO-BCS | 284 | 4.5 | 563 | 4.6 | 7,996 | 4.4 | 109,719 | 5.7 |
| ESTHER | 281 | 4.4 | 556 | 4.5 | 7,044 | 3.9 | 56,263 | 2.9 |
| KBCP | 198 | 3.1 | 396 | 3.2 | 5,803 | 3.2 | 35,505 | 1.8 |
| MCBCS | 575 | 9.0 | 2,301 | 18.7 | 34,689 | 19.3 | 500,659 | 25.8 |
| ORIGO | 374 | 5.9 | 1,154 | 9.4 | 18,464 | 10.3 | 349,260 | 18.0 |
| PBCS | 145 | 2.3 | 438 | 3.6 | 7,008 | 3.9 | 132,502 | 6.8 |
| RBCS | 292 | 4.6 | 728 | 5.9 | 9,188 | 5.1 | 71,588 | 3.7 |
| SBCS | 155 | 2.4 | 156 | 1.3 | 2,293 | 1.3 | 13,987 | 0.7 |
| SEARCH | 3,253 | 51.0 | 3,254 | 26.4 | 46,439 | 25.8 | 378,035 | 19.5 |
| Total | 6,378 |  | 12,326 |  | 180,172 |  | 1,939,984 |  |

**Supplementary table 2.** Estimated hazard ratios (HR) for all-cause mortality in 4,150 complete case breast cancer patients from multi-variable Cox proportional hazards.

| Variable | Cox model with ER evaluated by Citizen Scientists | | | Cox model with ER reported in BCAC data base | | |
| --- | --- | --- | --- | --- | --- | --- |
|  | HR | 95% CI | p-value | HR | 95% CI | p-value |
| Age | 1.00 | 0.99-1.00 | 0.812 | 1.00 | 0.99-1.00 | 0.752 |
| Stage |  |  |  |  |  |  |
| I |  |  | Ref |  |  | Ref |
| II | 2.33 | 1.89-2.88 | <0.001 | 2.32 | 1.88-2.87 | <0.001 |
| III | 5.27 | 3.94-7.07 | <0.001 | 5.27 | 3.93-7.07 | <0.001 |
| IV | 24.16 | 15.87-36.79 | <0.001 | 23.25 | 15.24-35.47 | <0.001 |
| Grade |  |  |  |  |  |  |
| 1 |  |  | Ref |  |  | Ref |
| 2 | 1.67 | 1.28-2.17 | <0.001 | 1.63 | 1.25-2.13 | <0.001 |
| 3 | 2.63 | 2.01-3.42 | <0.001 | 2.51 | 1.91-3.30 | <0.001 |
|  |  |  |  |  |  |  |
| ER positive | 0.21 | 0.14-0.31 | <0.001 | 0.22 | 0.16-0.31 | <0.001 |
| ER positive TVC* | 1.27 | 1.18-1.37 | <0.001 | 1.26 | 11.18-1.33 | <0.001 |

*****time varying covariate
